# Structure-based Design of JOC-*x*, a Conjugatable Tumor Tight Junction Opener to Enhance Cancer Therapy

**DOI:** 10.1038/s41598-019-42229-3

**Published:** 2019-04-16

**Authors:** Ragan Pitner, Jiho Kim, Jenn Davis-Bergthold, Cheri Turner, Emilie Vassal-Stermann, Hongjie Wang, Jaclyn Adams, Lauren Carter, Jeffrey A. Ahlgren, Pascal Fender, André Lieber, Darrick Carter, Sean A. Gray

**Affiliations:** 1 PAI Life Sciences, Inc., Seattle, WA USA; 2 University of Washington, Dept. of Immunology, Seattle, WA USA; 3 University of Washington, Program in Pathobiology, Dept. of Global Health, Seattle, WA USA; 4 Institut de Biologie Structurale, UMR5075, CNRS/CEA/UGA, Grenoble, France; 5 University of Washington, Division of Medical Genetics, Seattle, WA USA; 6 Institute for Protein Design, Department of Biochemistry, University of Washington, Seattle, WA USA; 7 Wyatt Technology Corporation, Santa Barbara, CA USA; 8 Compliment Corp., Seattle, WA USA

## Abstract

Disorganized intercellular junctions are critical for maintaining the integrity of solid epithelial tumors and prevent the infiltration of oncological therapies into the bulk of the malignancy. We have developed small, recombinant proteins which bind a critical junction protein, desmoglein 2, triggering the transient and specific opening of tumor tight junctions allowing for infiltration of the tumor with immune cells, oncolytic viruses, drugs, and other therapeutics. Our new molecule, JOC-*x*, is a promising candidate for a new class of tumor-targeting agents that accumulate both around and within tumors and remodel the tumor microenvironment. Native cysteines were removed from the parental protein, JO-4, followed by addition of a single cysteine to allow for convenient attachment of various payloads that can be targeted directly to the tumor. Our tumor-targeting protein exhibits high avidity, minimal aggregation, and is easily purified at good yields from *E. coli*. For proof of concept, we demonstrate effective conjugation to biotin as a model for flexible co-targeting, addition of metal ion chelators as models for imaging and radiotherapy, and linkage of the TLR3 agonist poly(I:C) as a model immune-oncologic agent. This second-generation cancer co-therapeutic protein is optimized for activity and primed for cGMP manufacture in preparation for upcoming clinical studies.

## Introduction

There are physical barriers to tumor penetration by cancer drugs. One of the key features of epithelial tumors is the presence of intercellular junctions that link cells to one another and act as barriers to the penetration of molecules with molecular masses greater than four hundred Daltons^1–3^. Several studies have shown that up-regulation of epithelial junction proteins correlate with increased resistance to treatment - including with therapies using monoclonal antibodies and chemotherapeutics^4–6^. One of these junction proteins, desmoglein 2 (DSG2), is a membrane glycoprotein which participates in the formation of tight junctions. Many epithelial cancers are known to highly upregulate the production of DSG2 resulting in formation of a network of cellular “staples” reminiscent of poorly organized junctions that render the tumors resistant to permeation by immune cells and cancer treatments. In one study, levels of DSG2 were found in all of the 60 primary and metastatic breast cancer biopsies analyzed^7^. Moreover, mRNA profiling of ovarian cancer biopsies revealed overexpression of *dsg2* message in at least 50 samples analyzed (data not shown). It is thought that the epithelial phenotype of cancer cells and their ability to form physical barriers provides protection from the host immune system and/or elimination by cancer therapeutics^8^.

**Figure 1:**
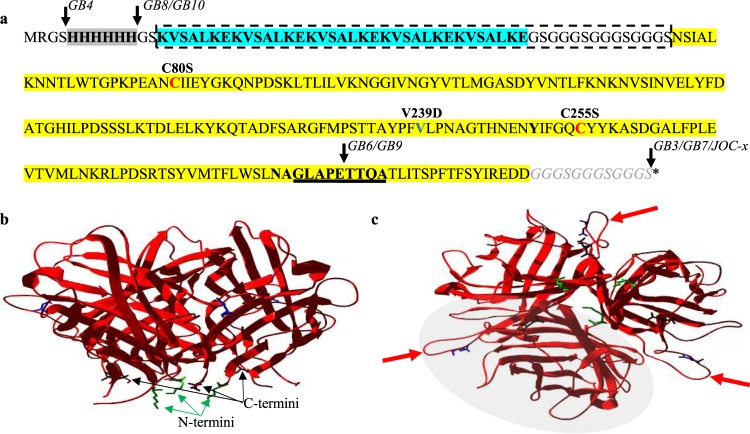
Sequence and structure of JO-1. Panel (a) shows the amino acid sequence of JO-1 with the 6× His-tag highlighted in grey, the dimerization domain is shown in bold text and highlighted in light blue, and the Ad3 knob highlighted in yellow. The affinity-enhancing V239D mutation and two internal cysteines at aa80 and aa255 are indicated above their respective residue. The H-I loop is underlined. In dimerization domain-deleted mutants, residues deleted are shown by the dashed box. Cysteinyl residues were inserted at locations shown by black arrows in each GB protein indicated next to the arrow. Panel (b) represents side view of the JO-4 trimer structure indicating the location of the N- and C-termini. Panel (c) shows a top down view of the trimer with red arrows highlighting the DSG2 binding loop in this view. The grey shaded oval approximates one JO-1 monomer. The structures are adapted from that of PDB Accession # 1H7Z_A as reported previously^11,12^.

Junction Opener 1 (JO-1) is a protein that binds to DSG2 and preferentially opens tumor tight junctions^7^ (Fig. 1). JO-1 was derived from the C-terminal knob domain of protein fibers on Adenovirus serotype 3 (Ad3) capsids, which mediates binding of the virus to DSG2 within the tight junctions. This binding initiates a cascade of events within the host cell leading to DSG2 shedding and eventual opening of tight junctions, thus allowing for translocation of the virus between epithelial cells through the basolateral intercellular space^9^. This phenomenon of opening tight junctions provided the basis for developing a co-therapeutic protein to enhance entry of biologics and chemotherapeutic agents such as Doxorubicin, Abraxane, or Irinotecan into tumors. In healthy cells, DSG2 is sequestered to the lateral cell junctions and is minimally available for binding. However, depolarization of tumor cells during epithelial to mesenchymal transition (EMT) leads to localization of DSG2 protein throughout the tumor rather than only within the cell junctions. Additionally, this is coupled with increased DSG2 expression providing an attractive target for our junction openers.

Early attempts at exploiting this property of Ad3 included recombinant expression of the fiber knob domains responsible for DSG2 binding (shown in yellow in Fig. 1a). However, these proteins did not produce the desired tight-junction opening effects possibly due to inability of the knob protein to form active higher-order quaternary structures^10,11^. To overcome this obstacle, a dimerization domain (DD) was added to facilitate multimerization^10^. This domain, a K-coil, is comprised of 5 repeats of 7 amino acids (shown in blue in Fig. 1a). Upon addition of the DD to the knob protein derived from Ad3, the resulting recombinant protein (JO-1) was able to form multimers, bind DSG2 *in vitro*, and inhibit Ad3 virus entry into HeLa cells. The JO-1 protein has since been extensively characterized, including the solved crystal structure of the active trimer (Fig. 1b,c). The trimeric form of the protein is the minimal conformation which will bind DSG2, although with low affinity^11^. However, for junction-opening activity, higher-order multimers are required, including the trimer-dimer, which has been described as a barbell structure^10,11^.

Mutagenic libraries of Ad3 fiber knobs identified a panel of mutations conferring higher affinities to DSG2 and enhancing viral inhibition in a functional assay^11^. DSG2 binding by select mutants was measured by surface plasmon resonance (SPR) to determine if the affinity increase was due to faster association rates or more stable dissociation rates^11^. One mutation, substituting the native valine to an aspartic acid at position 239 (V239D), resulted in a nearly 1000-fold increase in affinity as measured by a shift of the dissociation constant (K_D_) from 10.8 µM for the original JO-1 to 11.4 nM for the V239D mutant (designated JO-4)^11^. The JO-4 V239D mutation is localized to a region of the knob corresponding to a prominent loop (E-F) known to be critical for stabilizing DSG2 binding (indicated with red arrows in Fig. 1c). An additional eight Ad3 fiber knob mutations in three key regions (C-D loop, F-G loop, and H-I loop) were identified that reduced or ablated DSG2 binding^11^. One mutation in particular, E299V, located in the H-I loop region (underlined in Fig. 1a), was shown to reduce DSG2 binding in the mutant by 80%^11^.

Preliminary studies in mice have shown these junction openers to be effective at enhancing cancer therapy and very well tolerated even at high doses in non-human primates^12^. This promising *in vivo* data has resulted in an application as an investigative new drug (IND) for co-therapeutic treatment of certain types of tumors^12^. While co-administration of the JO-4 with effector molecules is an attractive model with substantial scientific support, the ability to produce a conjugatable junction opener with a specific site for covalent attachment to effector molecules would confer further production and deployment advantages. We envision that our designer junction openers would enable nanoparticle delivery to multiple cancers/tumors and may also be useful for chimeric antigen receptor (CAR) T-cell targeting. With respect to cancers, depolarization of cells during EMT as well as upregulation in DSG2 expression in tumors relative to normal tissues makes for an attractive target for our molecules.

Our lead JO-4 derivative, termed JOC-*x* (for Junction Opener Conjugated to *x*), could be directly attached to tumor imaging molecules such as europium or further conjugated with radionuclides for direct ionization of the tumor microenvironment. We investigated direct conjugation of polyriboinosinic:polyribocytidylic acid [poly(I:C)] to JOC-*x* for targeting and activating TLR3 and MDA5 receptors. By targeting poly(I:C) directly to the tumor-resident dendritic cells (DC), we would mimic natural infection by dsRNA viruses and initiate a robust inflammatory response typified by recruitment and activation of CD8+ T cells. Activation of TLR3 on tumor cells can also lead to tumor killing by initiating apoptosis, as has been demonstrated with the HPV vaccines BiVax and TriVax^13,14^. Moreover, numerous cancers are characterized by upregulation of TLR3, a receptor for poly(I:C), and in most of these cancers this TLR3 positivity is predictive of a favorable outcome if poly(I:C) is used as an immune therapeutic^13–18^. Given the ability of JOC-*x* to specifically localize to tumors overexpressing DSG2 and the known anti-tumor effects of poly(I:C), we envision that the JOC-poly(I:C) conjugate would exhibit enhanced targeting of tumors, increased potency, and overall less toxicity due to reduced binding to healthy tissues expressing TLR3 receptors and DSG2 that is properly sequestered in tight junctions.

We report here development, characterization, and *in vitro* validation of a new junction opening molecule, JOC-*x*. Preliminary characterization of JOC-*x* reveals many attractive features for production, manufacture, co-administration, and conjugated targeting making it a promising candidate to move towards human trials.

## Results

### Generation of a protein without internal cysteines and with a free cysteine for conjugation

JO-1 has two native cysteinyl residues (amino acids 80 and 255) which, based on the crystal structure, are spatially distant and unlikely to form intramolecular disulfide bridges. Ten JO-1 derivatives (termed GB1–10) were generated, with six containing the dimerization domain and four in which the dimerization domain was eliminated to interrogate the effects on multimerization and DSG2 binding activity. Supplementary Fig. 1 shows a pictorial representation of each GB protein compared to JO-1, with key changes highlighted. GB1 is a codon optimized protein identical to JO-1 but optimized to maximize expression in *E. coli*. GB2 was constructed by mutating the native internal cysteines of GB1 to serinyl residues to limit oxidation and covalent aggregation. Next, we reintroduced a single cysteine to act as a designer sulfhydryl-based site for targeted conjugation at various locations within the proteins as follows: in GB3 the cysteine was added at the C-terminus following a flexible glycine serine linker [(G_3_S)_3_]; in GB4 and GB10, the single cysteinyl residue was added at the N-terminus either before (GB4) or immediately following (GB10) the His tag; and for GB6, the cysteine was introduced proximal to the glutamic acid residue at position aa 299 within the H-I loop (PECTT, underlined in Fig. 1a), a key region known to stabilize DSG2 binding.

### The free cysteine functions as a covalent mediator of multimerization

The DD contains 5 repeats of seven amino acids, with two lysine residues per repeat, which we predicted could potentially result in ribosomal stalling during translation and lower protein yields. Addition of the DD, with its predicted isoelectric point (*p*I) of ~10.91, to the Ad3 knob results in an increase in predicted *p*I from ~7.32 (Ad3 knob) to ~8.65 (JO-1). We therefore hypothesized that removal of the DD would likely (1) lower aggregation beyond the levels observed with the cysteine-deleted GBs, (2) enhance protein expression levels, and (3) return the protein to a neutral *p*I leading to lower residual endotoxins following purification. To test this hypothesis, GB5, GB7, GB8 and GB9 were all designed without the DD and a single cysteine inserted as follows: GB5 contained no cysteine; GB7 was constructed similar to GB3 with the C-terminal (G_4_S)_3_ followed by a terminal cysteine residue; GB8 contained a cysteine immediately following the His tag; and GB9 was similar to GB6 with a cysteine in the H-I loop (Supplementary Fig. 1).

### Elimination of the dimerization domain confers multiple production advantages

Nine of the ten GB proteins produced in *E. coli*. The exception, GB6, failed to express stable protein and was eliminated from further analysis. Expression levels of GBs containing the DD ranged from 0.5–5 mg/L, similar to JO-1, whereas clones without the DD were 4–10-fold higher and ranged from 5–20 mg/L. Endotoxin levels of clones containing the DD ranged from 80,000 to ~1,500,000 EU/mg with an average of 691,300 EU/mg as determined using the Limulus Amebocyte Lysate (PTS-LAL) assay (Charles River Laboratories, Inc., Charleston, SC). In addition, endotoxin was very difficult to remove without significant protein losses. In contrast, endotoxin levels of DD-deleted clones were 100- to 1000-fold lower ranging from ~3000–20000 EU/mg. Production chromatography of GB7 (with no DD and a single C-terminal cysteine) resulted in an average endotoxin level of only 2500 EU/mg which was easily reduced to <10 EU/mg following a single pass through an endotoxin removal column.

### Functional responses are preserved

Each of the GB proteins migrated according to their expected molecular weight on boiled and reduced SDS-PAGE (Supplementary Fig. 2a). We assessed multimerization using samples that were not reduced and not boiled prior to SDS-PAGE (Supplementary Fig. 2b), which-while not true native analysis-allows this family of proteins to retain multimeric forms even in the presence of SDS detergent. This was done because previous data had indicated that denatured, improperly folded, or otherwise inactivated JO-1 does not form higher-order multimers on non-reduced SDS-PAGE, does not bind DSG2, and does not inhibit viral entry^11^. As shown in Supplementary Fig. 2b, several multimeric states were observed including monomers (M), trimers (T), trimer-dimers (T-D), and multimers/aggregates (Agg.) at >150 kDa. In the non-reducing gel, all nine GB proteins formed higher order multimers, although a significant percentage of GB9 maintained a monomeric state. GB5 appeared to form trimers but did not produce higher MW isoforms such as trimer-dimers.

To confirm the presence of active conformations, the panel was tested for binding to human DSG2 by Western analysis probing with labeled hDSG2 (Supplementary Fig. 2c). Except for GB5 and GB9, prominent DSG2 binding was visible in the region of the blot corresponding to trimers, timer-dimers, and aggregates, but not at the size corresponding to monomers. This result was not unexpected for GB5 given the lack of higher MW multimers beyond the trimer. GB9, despite forming multiple higher order multimers, failed to bind DSG2 confirming earlier reports on the importance of the H-I loop as one of three key regions required for DSG2 binding by the Ad3 knob^11,19^.

We next assessed the ability of the GBs to bind to DSG2 *in vitro* using HeLa cells in a competition-based viral inhibition assay (VIA) (Supplementary Fig. 2d)^11^. This assay determines the minimum protein concentration required to inhibit 50% entry of a fluorescent Ad3 reporter virus and is a robust predictor of *in vivo* activity. These viral inhibition curves can be fit to a 5-parameter curve from which the concentration at which 50% of viral entry is inhibited (IC_50_) can be calculated and reported in µg/mL. Results of DSG2 binding and IC_50_ values for the ten GBs are listed in Table 1. Seven of the nine GBs had binding curves similar to JO-1, with GB5 showing significantly lower and GB9 showing almost no Ad3 virus inhibition.

**Table 1:** Phenotype of JO4 derivatives.

Protein	DSG2 binding	VIA IC_50_ (µg/mL)
JO-4	Yes	0.067
GB1	Yes	0.025
GB2	Yes	0.030
GB3	Yes	0.029
GB4	Yes	0.037
GB5	Minimal	0.241
GB6	n/a	n/a
GB7/JOC-*x*	Yes	0.032
GB8	Yes	*0.012*
GB9	No	>10
GB10	Yes	0.026

### Down-selection of next generation conjugatable junction openers

Based on production levels, DSG2 binding, and viral inhibition results, we down-selected GB3 and GB7 for further characterization of multimerization. To improve binding kinetics of GB3 and GB7, we introduced the JO-4 mutation by substituting a valine at position 239 for an aspartic acid resulting in the high affinity versions GB3_V239D_ and GB7_V239D_. We prioritized GB7 over GB8 since having a cysteine at the C-terminus would be advantageous for conjugation of the protein to effector molecules (drugs, antibodies, poly(I:C), or isotopes) as well as delivery systems such as nanoparticles. With respect to GB8, we also hypothesized that conjugation to a cysteine proximal to the 6× his tag could pose steric hindrances which could affect downstream purification efforts.

Domains of DSG2 are cleaved and shed by the action of matrix metalloproteinases in a process that is enhanced by treatment with our junction opener constructs. This shed DSG2 can be quantified using a simple ELISA^20^. Over the course of 48 hours, JO-4, GB3_V239D_, or GB7_V239D_ all resulted in increased DSG2 shedding compared to untreated cells (data not shown). For a more accurate measurement of DSG2 binding, we compared the avidities and association/dissociation rates of JO-x derivatives to DSG2. Surface plasmon resonance (SPR) analysis was performed by immobilizing recombinant human DSG2 (rhDSG2) onto a Biacore sensor chip and flowing JO-4, GB3_V239D_, or GB7_V239D_ across the surface. A roughly 20-fold more avid binding of DSG2 was determined for GB7_V239D_ compared to JO-4 (0.58 nM and 11.4 nM, respectively). This apparent higher affinity of GB7_V239D_ appeared to be due to both a faster association rate and slower dissociation rate compared to JO-4 (Table 2a). Of the three proteins, GB3_V239D_ had the highest affinity of 0.11 nM primarily due to a nearly one log faster association rate of 4.4 × 10^6^ M/s compared to JO-4 and nearly 3-fold faster on-rate compared to GB7_V239D_. In this format, both JO-4 and the GB3_V239D_ proteins appeared to bind to the sensor surface somewhat non-specifically and were more difficult to regenerate from the sensor surface compared to GB7_V239D_. We hypothesized that presence of positively charged residues within the DD of JO-4 and GB3_V239D_ may have resulted in binding to the negatively charged sensor surface and that the greater abundance of multimers and aggregates, more prominent in JO-4 and GB3_V239D_, may have retarded diffusion of these molecules compared to GB7_V239D_. To minimize non-specific binding that may be due to the DD, we repeated the assay by immobilizing one protein containing the DD (JO-4) and one without the DD (GB7_V239D_) while flowing DSG2 across the surface (GB3_V239D_ was not tested in this format). In this format, the apparent avidity of JO-4 to DSG2 improved from 11.4 nM to 0.58 nM, while the affinity of GB7_V239D_ for DSG2 was nearly identical albeit slightly reduced (Table 2b).

**Table 2:** Biacore analysis of binding to hDSG-2.

Protein	k_a_ (M/s)	K_d_ (1/s)	K_D_ (nM)
**a. Rates following immobilization of hDSG2 protein**			
JO4	2.32 × 10^5^	2.64 × 10^−3^	11.4
GB3_V239D_	**4.4 × 10** ^**6**^	5.0 × 10^−4^	**0.11**
GB7_V239D_/JOC-*x*	1.4 × 10^6^	8.3 × 10^−4^	0.58
**b. Rates following immobilization of junction opener proteins**			
JO4	7.9 × 10^5^	**4.6 × 10** ^**−4**^	0.58
GB3_V239D_	Not tested		
GB7_V239D_/JOC-*x*	6.3 × 10^5^	7.5 × 10^−4^	1.19

### Junction openers without the dimerization domain exhibit minimal aggregation

**Figure 2:**
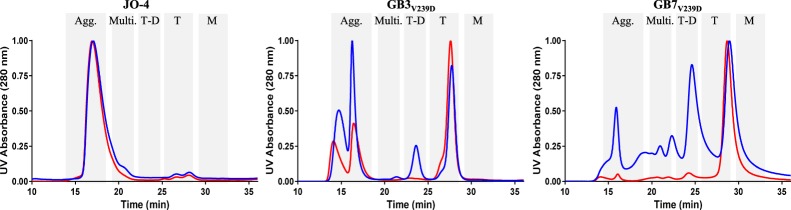
Distribution of protein multimerization states determined by SEC analysis. The SEC plots for JO-4 (left), GB3_V239D_ (middle), and GB7_V239D_ (right). The blue line represents the A280 absorbance for the oxidized protein while the red line represents the reduced protein. The *y*-axis represents the approximate fraction of each peak while the *x*-axis shows the elution time in minutes. The grey shaded boxes indicate the approximate retention time for (Agg) aggregates, (Multi) multimers, (T-D) trimer-dimers, (T) trimers, and (M) monomers based on MALS analysis.

To investigate the quaternary structure of the proteins, we performed size-exclusion chromatography and multi-angle light scattering analysis (SEC-MALS) on JO-4, GB3_V239D_, or GB7_V239D_. Each protein was tested in the native (presumably oxidized) form or following treatment with low levels of dithiothreitol (DTT) to reduce disulfide bonds. The UV traces of these analyses are shown in Fig. 2 whereby the shaded and labeled boxes indicate where the aggregates (Agg.), multimers (Multi.), trimer-dimers (T-D), trimers (T), or monomers (M) eluted based on size determination by the MALS detector.

The MALS analyses - including percentage of each species, molecular weights, and elution times - are summarized in Table 3. In its oxidized state, JO-4 presented a dominant peak in the region corresponding to aggregates with approximately 94% existing as aggregates or multimers and a minority existing as monomers or trimers. Less than 1% existed as trimer-dimers. Reduction of JO-4 with DTT did not significantly alter the aggregation phenotype. Removal of internal cysteines in GB3_V239D_ significantly reduced the aggregation phenotype with 57% of the protein existing as aggregates or multimers, 10% existing as trimer-dimers, and 33% existing as trimers. Reduction of GB3_V239D_ resulted in a slight reduction in aggregates, a 3-fold reduction in trimer-dimers, and significant increase of trimers from 33% to 53%. In the oxidized state, GB7_V239D_ existed as trimers (34%) and 31% as trimer-dimers, with 35% existing as multimers, or aggregates. Upon reduction, the percentage of trimers significantly increased (89%) with a concomitant reduction in all three higher order species to a total of 10%. This is consistent with the engineered cysteinyl residue performing as expected allowing for targeted multimerization which can be reversed by reduction. For all 3 proteins, only a small amount was found in their monomeric form even after reduction. This agrees with un-boiled, un-reduced SDS-PAGE analysis of GB7 (Supplementary Fig. 2b) in which almost no visible monomers are observed even in the presence of reducing and denaturing agent.

**Table 3:** Distribution of protein multimerization states determined by MALS analysis.

Approx. Elution Time (min)	Approx. MW (kDa)	Likely species	% total of each species					
			JO4		GB3_V239D_		GB7_V239D_/JOC-*x*	
			Oxidized	Reduced	Oxidized	Reduced	Oxidized	Reduced
13.8–18.2	3000-578	Aggregate (Agg.)	90	94	55	43	12	4
19.2–21.5	334-289	Multimer (Multi.)	4	<0.5	2	<0.5	23	4
22.2–24.6	167-144	Trimer-Dimer (T-D)	<0.5	1	10	3	31	2
25.0–28.3	83-72	Trimer (T)	2	2	33	53	34	89
29.2–32.4	24–28	Monomer (M)	4	3	<0.5	<0.5	<0.5	<0.5

**Figure 3:**
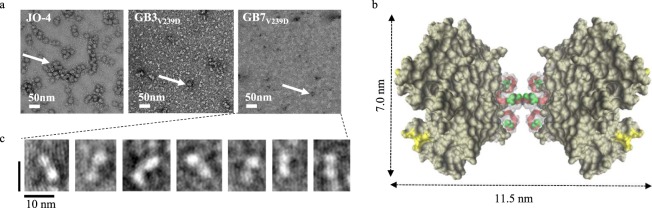
TEM images of protein aggregation and trimer-dimer formation. Panel (a) uranyl acetate negative staining of protein for JO-4 (left), GB3_V239D_ (middle), and GB7_V239D_ (right). The white arrows indicate aggregates which appear nearly 50 nM in diameter according to the 50 nM size bar in white at the lower left of the figure. Panel (b) the space-filling model of the trimer-dimer of JO-4 was created in VMD 23 using the known x-ray crystallographic coordinates of JO-4. The model depicts a barbell shape with an approximate length of 11.5 nm and a width of 7 nm. Panel (c) fine resolution enhancement images of white specks seen in the GB7_V239D_ image in panel a. The images reveal structures resembling the barbell structure in both size and shape of the modeled trimer-dimer.

Further investigation of the quaternary structure was performed using uranyl acetate negative staining of protein smears and imaging by transmission electron microscopy (TEM)^21^. As shown in Fig. 3a, JO-4 exhibited relatively uniform higher order structures approaching 50 nm in diameter (white arrows) that resemble the penton-dodecahedra (Pt-Dd) described previously^9,22^. Penton-dodecahedra are believed to be made up of 12 pentons of pentameric base and 12 trimers of the fiber knob with a predicted mass of at least 1253 kDa. This is similar to the size measured by MALS for the JO-4 aggregates, estimated to average 1120 kDa in the oxidized state. By TEM, GB3_V239D_ exhibits fewer high MW aggregates compared to JO-4. This observation is even more pronounced with GB7_V239D_ as only a few aggregates are observable in the image field. The white specks in the background and prevalent throughout the images were presumed to be trimers and/or trimer-dimers, the latter of which have been described as resembling barbell-like structures. Using the Visual Molecular Dynamics program (VMD, U. of Illinois Urbana-Champaign)^23^ and the known x-ray crystallographic coordinates for JO-4, we modeled the trimer-dimer structure as shown in Fig. 3b. The resulting trimer-dimer structure indeed appears barbell-shaped with approximate dimensions of 7 nm wide by 11.5 nm long. Fine resolution enhancement of seven of these white specks from the GB7_V239D_ image are shown in Fig. 3c. These specs clearly resembled the barbell-shape of the modeled trimer-dimer (Fig. 3b) in both size and shape.

### Junction openers with alternate multimerization strategies maintain full viral inhibition

As mentioned above, SEC-MALS analysis suggested that reduction had almost no impact on JO-4 multimerization, minimal impact on GB3_V239D_ multimerization, and a pronounced impact on GB7_V239D_ multimerization. It has been shown that the junction openers will only open tight junctions or inhibit viral entry as a trimer-dimer, multimer, or aggregate^10,11^. Moreover, trimers, but not monomers, will bind DSG2 but will not open tight junctions^10,11^. We therefore sought to test the ability of JO-4, GB3_V239D_, and GB7_V239D_ to inhibit Ad3 viral entry in the reduced or oxidized state. Based on SEC-MALS analysis, we predicted that JO-4 would show equivalent levels of viral inhibition given that it maintains higher order structures following reduction. However, we expected GB7_V239D_ to exhibit the greatest loss in viral inhibition given that MALS analysis showed that ~89% of the protein was found as trimers following reduction. Results of the viral inhibition assay on reduced and non-reduced proteins are shown in Supplementary Fig. 3a while the IC50 values are shown in Supplementary Fig. 3b. As expected for JO-4, oxidized and reduced inhibition curves were nearly indistinguishable from one another and resulted in nearly identical IC_50_ values. For GB3_V239D_, reduction resulted in an approximately two-fold decrease in viral inhibition likely due to loss of some aggregates and the three-fold reduction in trimer-dimers estimated by MALS analysis. As expected with GB7_V239D_, reduction with DTT resulted in a 5-fold decrease in viral inhibition. This was likely due to the 4-fold loss of aggregates and multimers (from 35% to 8%) and the 15-fold loss of trimer-dimers (from 31% to 2%) upon reduction as measured by MALS analysis (Table 3).

### Conjugation of JOC-*x* with novel payloads

**Figure 4:**
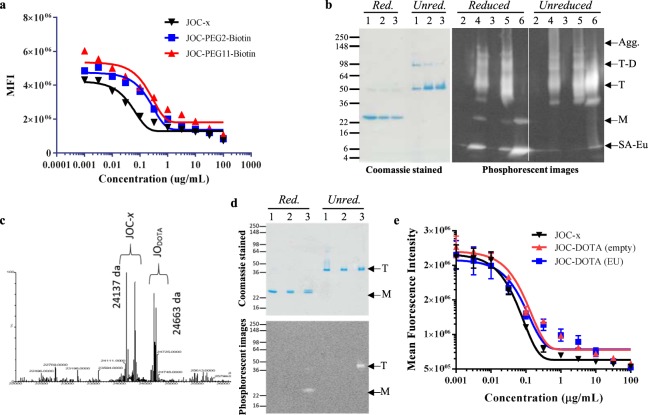
Conjugation of JOC-*x* with PEG-biotin or DOTA-EU. Panel (a) Shows the viral inhibition curves for JOC-*x* before and after conjugating to PEG2- or PEG11-biotin. Panel (b) JOC-*x* and JOC-Biotin conjugates were mixed with streptavidin-europium (SA-Eu) and resolved by SDS-PAGE with or without boiling or reduction by DTT. One gel was stained with Coomassie to visualize formation of multimers and one gel transferred to a PVDF membrane and imaged using a Spectramax I3x Imager to visualize Eu phosphorescence at an emission spectrum of 616 nm. Multimeric forms are indicated to the right of the gel/blot whereby SA-Eu = free streptavidin-europium, M = Monomer, T = Trimers, T-D = Trimer-Dimers, and Agg. = Aggregates/multimers. Numbering of lanes is as follows: 1 = JOC-*x*, 2 = JOC-PEG2-biotin, 3 = JOC-PEG11-biotin, 4 = JOC-PEG2-biotin-SA-Eu, 5 = JOC-PEG11-biotin-SA-Eu, 6 = SA-Eu alone. Panel (c) A mass spectrometry spectrum for JOC-DOTA is shown indicating the position of unconjugated JOC-*x* (24137 Da) and JOC-DOTA (24663 Da). Panel (d) Unconjugated JOC-*x* (1), JOC-DOTA (empty) (2), and JOC-DOTA (Eu) (3) were resolved by SDS-PAGE, with or without boiling or reduction by DTT. One gel was stained with Coomassie (top gel) to visualize formation of multimers and one gel transferred to a PVDF membrane and imaged using a Spectramax I3x Imager (lower blot) as described. Positions of Trimers “T” and monomers “M” are noted to the right of the gel/blot. Panel (e) Viral inhibition curves of JOC-*x*, JOC-DOTA (Empty), and JOC-DOTA (Eu) are shown indicating that DOTA-Eu conjugation only minimally reduces DSG2-mediated viral entry.

Based on the biochemical and biophysical characterization of JO-4, GB3/GB3_V239D_, and GB7/GB7_V239D_, we selected GB7_V239D_ as our lead molecule and renamed it JOC-*x* for Junction Opener Conjugated to *x* to better describe its functional attributes. We then sought to demonstrate the utility of JOC-*x* as a reagent for pre-targeted radioimmunotherapy (PRIT), direct tumor imaging, or immune therapy^24–26^. The basis of PRIT involves injection of a biotinylated tumor-targeting molecule, allowing the molecule to bind its target, and then forming a complex at the site of the tumor following a second injection of streptavidin conjugated to either an imaging agent or radionuclide. We biotinylated JOC-*x* at the terminal cysteinyl residue using the EZ Link Maleimide-PEG2 Biotin or PEG11 Biotin kits (Thermo Scientific, Rockford, IL.). The purpose for the PEG2 or PEG11 spacers was to assess whether biotinylation would impair ability of the monomeric protein to form bioactive multimers, and if so, would increasing the linker length reduce steric hindrance and increase multimerization and activity. Following biotinylation, the JOC-*x* and JOC-PEG2/PEG11 conjugates were tested for viral inhibition as described (Fig. 4a). JOC-PEG2-biotin and JOC-PEG11-biotin both inhibited virus with only slightly impaired IC_50_ values of 0.125 and 0.203 μg/mL, respectively, compared to 0.032 μg/mL for the unconjugated JOC-*x*. As shown in the Coomassie stained panel in Fig. 4b, conjugation of either PEG2- or PEG11-biotin did not impair the ability of JOC-*x* to form multimers as evidenced by the formation of the bands in the Trimer (T) and Trimer-Dimer (T-D) range of the unreduced samples. To further functionalize the JOC-PEG-biotin conjugates, we added a streptavidin-europium (SA-Eu) moiety to the conjugate and analyzed their ability to form trimers and trimer-dimers even with the bulky SA-Eu groups attached (Fig. 4b). Europium is a rare earth metal and was chosen for two reasons: (1) it is often used in *in vivo* imaging due to its phosphorescent properties; and (2) it is a non-radioactive surrogate for radionuclides that may later be used for direct imaging or irradiation of tumors. The phosphorescent panel in Fig. 4b shows that even with successful loading of SA-Eu to both PEG2 and PEG11 biotin conjugated JOC-*x* they were able for form trimers, trimer-dimers, and aggregates (indicated by *T*, *T-D*, and *Agg* on Fig. 4b). An inhibition assay with the JOC-PEG-SA-Eu conjugates resulted in atypical curves of viral inhibition that could not be fit with our typical 5-parameter fit. This is likely due to multiple binding interactions taking place simultaneously between SA multimers, SA-JOC-PEG-biotin, and JOC-PEG-SA-Eu to the DSG2 protein on cells. However, we do not consider this problematic given that the full conjugate-payload, JOC-PEG-SA-Eu, would not be directly injected into an animal, but rather would be used stepwise (as described above for PRIT).

A different methodology to generate a directly labeled, less bulky, and more flexible tumor imaging and radiotherapy agent involved conjugation to 1,4,7,10-tetraazacyclododecane-1,4,7,10-tetraacetic acid (DOTA) to the JOC-*x* resulting in JOC-DOTA. DOTA/Eu labeled proteins have been used extensively for *in vivo* imaging^27–29^ and also have the advantage of being able to sequester radionuclides, such as yttrium-90, which may be used to irradiate their targets^30–33^. Because of the small size of DOTA (526 Da), we used mass spectrometry to confirm that the DOTA molecule was attached to JOC-*x*. Figure 4c shows a mixture of unconjugated JOC-*x* and JOC-DOTA with their respective spectra at 24137 Da and 24663 Da, respectively.

The JOC-DOTA was then complexed with europium ions to form JOC-DOTA (Eu) and successful labeling was demonstrated by reduced and non-reduced SDS-PAGE (Fig. 4d). Coomassie staining (top gel) showed that JOC-*x*, JOC-DOTA (empty), and JOC-DOTA (Eu) all were able to form trimers in the unreduced analysis. A second, identical gel was transferred to a PVDF membrane and imaged using a Spectramax I3x Imager (lower blot) to capture europium phosphorescence. As shown in Fig. 4d, only the JOC-DOTA (Eu) produced phosphorescence at the monomer (M) position in the reduced gel and the trimer (T) position in the unreduced gel. We demonstrated that the DOTA conjugates maintained their ability to bind DSG2 using the viral inhibition assay (Fig. 4e) with IC_50_ values of 0.055, 0.131, and 0.254 μg/mL for JOC-*x*, JOC-DOTA, and JOC-DOTA (Eu), respectively.

### Viral inhibition and TLR3 signaling following poly(I:C) conjugation to JOC-*x*

**Figure 5:**
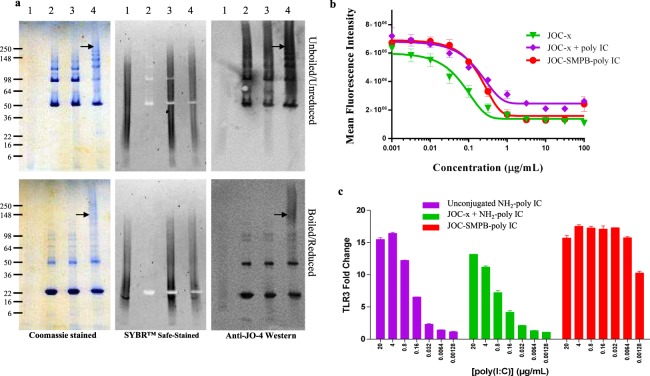
Viral inhibition and TLR3 signaling mediated by JOC-poly(I:C). Panel (a) unboiled/non-reduced and boiled/reduced SDS-PAGE analysis of 1-unconjugated poly(I:C), 2-unconjugated JOC-*x*, 3-a mixture of unconjugated poly(I:C) and JOC-*x*, and 4-JOC-poly(I:C) are shown following imaging by Coomassie staining (left), staining with the DNA intercalating dye SYBR Safe (middle), or following blotting to PVDF and reacting with anti-JO-4 antibodies by Western blot (right). The black arrow denotes the formation of a high MW complex at around 250 kDa in lane 4 (JOC-poly(I:C) conjugates) of all three panels proving the successful conjugation of poly(I:C) to JOC-*x*. Panel (b) viral inhibition curves of unconjugated poly(I:C). The *x*-axis represents the concentration of JOC protein in µg/mL while the *y*-axis represents the mean fluorescence intensity resulting from entry of reporter virus. A mixture of unconjugated poly(I:C) and JOC-*x*, and the JOC-poly(I:C) conjugate are shown indicating that the JOC-poly(I:C) was able to inhibit DSG2-mediated viral entry, albeit at a slightly lower concentration; Panel (c) shows the fold TLR3 activation mediated by binding of unconjugated NH_4_-poly(I:C) (purple bars), a mixture of unconjugated NH_4_-poly(I:C) and JOC-*x* (green bars), or JOC-poly(I:C) (red bars). Cells were incubated with 7 concentrations of agonist ranging from 20 μg/mL down to 0.00128 μg/mL in five-fold increments.

A final demonstration of the utility of JOC-*x* was generation of a conjugate that would lead to innate signaling and activation through the viral RNA sensors (TLR3 and MDA5) triggered by the synthetic ligand poly(I:C). Poly(I:C) was conjugated to JOC-*x* using the heterobifunctional crosslinker SMPB [succinimidyl 4-(p-maleimidophenyl) butyrate] linking the free cysteine on JOC-*x* to the amino terminus of poly(I:C). When conjugated to JOC-*x*, the poly(I:C) was expected to add an additional 120 to 600 kDa to JOC-*x* based on the typical size of the poly(I:C) used here which varies between 0.2 and 1.0 kb in length. Successful conjugation of poly(I:C) to JOC-*x* is shown in Fig. 5a, in which the black arrows in Lane 4 of the panels identify the JOC-poly(I:C) conjugates which maintain their predictably high molecular weight and conformation even when boiled and reduced (lower 3 panels of Fig. 5a). This conjugated form is not visible in either the unconjugated JOC-*x* (Lane 2), unconjugated poly(I:C) (Lane 1), or in the unconjugated mix of JOC-*x* and poly(I:C) (Lane 3) of Fig. 5a. Figure 5b shows the viral inhibition of unconjugated JOC-*x* (green line, triangles), an equimolar mixture of JOC-*x* and poly(I:C) (purple line, diamonds), and the JOC-poly(I:C) conjugate (red line, circles). The unconjugated mixture of JOC-*x* and poly(I:C) was included to measure direct binding of poly(I:C) to HeLa cells given that this cell line has been demonstrated to be TLR3 positive, although TLR3 is primarily found in endosomal compartments and MDA5 in intracellular^34^. In the presence of poly(I:C), unconjugated JOC-*x* exhibited a ~3.9-fold loss of viral inhibition suggesting possible binding competition. JOC-poly(I:C) conjugates maintained their ability to bind DSG2 and inhibit viral entry, although the slight shift of the inhibition curve to the right is reflective of an approximately 6.3-fold higher IC_50_ of the conjugate relative to the unconjugated protein (0.032 vs 0.203 μg/mL, respectively). To assess whether the JOC-poly(I:C) could bind to TLR3 receptors and induce cell signaling, we utilized an *in vitro* HEK-Blue human TLR3 reporter cell line (Invivogen, San Diego, CA). As shown in Fig. 5c, we demonstrated that JOC-poly(I:C) conjugates were able to bind to TLR3 and induce signaling at significantly lower protein concentrations compared to poly(I:C) alone or a mixture of JOC-*x* and poly(I:C). For example, with poly(I:C) alone (purple bars) or a mixture of JOC-*x* and poly(I:C) (green bars), the two highest concentrations (20 and 4 μg/mL) resulted in ~15 to 17-fold upregulation in NF-kB-phosphatase expression, but at the lowest two concentrations (0.0064 and 0.00128 μg/mL) only about a twofold upregulation was seen. Strikingly, at 0.032 μg/mL, the JOC-poly(I:C) conjugates (red bars in Fig. 5c) still exhibited ~18-fold upregulation compared to either poly(I:C) alone or unconjugated JOC-*x*. In fact, JOC-poly(I:C) maintained >18-fold upregulation at every concentration tested down to 0.0064 μg/mL, and even at 0.00128 μg/mL still induced a >10-fold increase in TLR3 signaling. These results suggest that the JOC-poly(I:C) conjugates may be significantly more potent in stimulating TLR3 signaling than poly(I:C) alone.

## Discussion

Epithelial cancers are characterized by tight junctions that create obstacles to naturally occurring immune cells, antibodies, and drug therapies as well as helping maintain the tumor microenvironment. We have described a new approach to exposing tumors to the action of the immune system and oncological therapy using designer proteins that bind to DSG2 and open tumor tight junctions. Our first generation molecule, JO-1, has been extensively characterized as a co-therapeutic for cancer treatment^7,35^. Binding by JO-1 leads to DSG2 cleavage, shedding, and activation of intracellular signaling pathways which facilitate the opening of junctions. JO-4, a high affinity version of JO-1, has been shown to be well tolerated *in vivo* and is being developed as a cancer co-therapeutic.

While producing a conjugatable version of JO for targeted cancer therapy we enhanced expression levels through codon optimization and removal of the dimerization domain. We mutated the internal cysteines to serines and found that this did not affect-and may even have enhanced-the activity of the protein as determined by DSG2 binding and viral inhibition. Restoration of a single cysteine was generally sufficient to recover DSG2 binding given that GB5, lacking both cysteines and the DD, failed to form higher MW isoforms and did not bind DSG2, yet still inhibited viral entry - albeit at a much lower level. Both GB7 and GB8, which differ from GB5 by only one cysteine, inhibited viral entry at comparable levels relative to JO-1. As expected, perturbation of the H-I loop involved in DSG2 binding ablated activity. We conclude that the single cysteine may lead to at least trimer-dimer formation resulting in recovery of DSG2 binding. We next down-selected GB3 and GB7 for further analysis of multimerization and aggregation, introduced the affinity-enhancing V239D mutation, and confirmed that the next generation clones GB3_V239D_ and GB7_V239D_ had retained comparable viral inhibition activity relative to JO-4. SPR analysis confirmed the association and dissociation rates and resulting affinities of GB3_V239D_ and GB7_V239D_ to hDSG2 were comparable or improved relative to JO-4.

SEC-MALS and TEM analysis of the aggregation state of the proteins revealed that JO-4 exists mainly as higher order structures resembling penton-dodecahedra (Pt-Dd) in both mass and shape. Reduction of JO-4 only minimally disrupted formation of these structures. Removing the cysteines (GB3_V239D_) and dimerization domains (GB7_V239D_) while adding a single terminal cysteine changed the aggregation state of the molecule while providing for an active molecule with the potential for directed attachment of therapeutics. We demonstrated that JO-4, GB3_V239D_, and GB7_V239D_ exhibited equivalent viral inhibition in the oxidized state, and that only GB7_V239D_ showed a marked reduction in viral inhibition upon reduction consistent with the idea that multimerization beyond trimers is required for activity and that the added cysteine provides for this ability.

TEM images of GB7_V239D_ revealed very few higher order structures relative to JO-4 as well as the presence of white specks throughout the image. Enhancement of these specks revealed that they were likely trimer-dimers, matching both the shape and predicted size of the trimer-dimers. Presence of these trimer-dimers combined with the comparable viral inhibition of GB7_V239D_ compared to JO-4 suggest that this protein is still fully able to bind DSG2 and act therapeutically.

Based on physical and biochemical characterization, we down-selected GB7_V239D_ for our conjugation experiments and renamed our lead molecule JOC-*x* to denote that it can open tumor junctions and be conjugated to a payload (*x*). Primary reasons for choosing GB7_V239D_ for our JOC-*x* included high production yields, low endotoxin binding, and controllable defined aggregation all while retaining similar DSG2 binding and viral inhibition relative to JO-4 (outlined in Table 4).

**Table 4:** Summary of key characteristics of JO4-derivatives.

Characteristic	JO-4	GB3_V239D_	GB7_V239D_/JOC-*x*
*p*I	8.91	9.11	*7.30*
Production level (mg/L)	1–5	1–3	*10–15*
Ave. endotoxin (EU/mg)	691,300 K	80,500	*2,500*
% aggregated by SEC^A^	90/94%	55/43%	*12/4*%
Affinity to DSG2 (nM)	0.58	*0.11*	1.2
DSG2 shedding (fold over PBS treated at 48 h)	1.44	*1.52*	1.39
VIA IC_50_ (μg/mL)	0.067	*0.029*	0.032

^A^*First value reported is untreated, second value is following reduction with DTT*.

As proofs-of-concept for this cancer-targeting platform we first sought to generate a reagent useful for pre-targeted immunotherapy (PRIT) and direct tumor imaging by adding pegylated biotins to JOC-*x*. These JOC-PEG-biotin conjugates were active in the viral inhibition assay and were able to bind to SA-Eu for potential use in PRIT applications.

To make a second radiotherapy molecule, we conjugated a metal chelation molecule, DOTA, to JOC-*x* and demonstrated effective labeling with free europium ions. The JOC-DOTA (Eu) chelates were able to bind DSG2 and inhibit virus *in vitro* suggesting they will bind to tumor cells *in vivo* and the metal chelate can be used to irradiate or mark the tumor for *in vivo* imaging.

Finally, we generated a potent JOC-poly(I:C) conjugate which also bound DSG2, although with a ~6.3-fold lower IC_50_ compared to JOC-*x* alone in our viral inhibition assay. The reason for the lower IC_50_ may be due to either impaired kinetics of the conjugate, possibly due to steric hindrances imposed by the high MW of the poly(I:C) or may be a competitive effect caused by poly(I:C) binding to TLR3 present in the HeLa cells themselves. Despite this lower level of binding in the VIA assay, the JOC-poly(I:C) induced 10- to 18-fold upregulation of TLR3 signaling using a HEK Blue TLR3 reporter cell line at every concentration tested down to 0.00128 μg/mL. In this assay, poly(I:C) alone only resulted in a 6-fold upregulation of TLR3 signaling at a concentration that was 125 times higher (0.16 μg/mL) than the JOC-poly(I:C). These results suggest that the JOC-poly-IC conjugates are significantly more potent in stimulating double stranded RNA sensor signaling than poly(I:C) alone. Given the dual-targeting of JOC-*x* to DSG2 and poly(I:C) to TLR3, we predict our JOC-poly(I:C) may exhibit dose sparing qualities by resulting in less non-specific binding of poly(I:C) to non-tumor TLR3 cells as well as targeting the host-directed therapeutic to tumors.

The goal of this study was to develop novel tumor targeting proteins that could be directly coupled to anti-tumor effector molecules. The lead molecule, JOC-*x*, exhibited enhanced biophysical properties with respect to production yields, aggregation, and low residual endotoxins following purification while maintaining high affinity binding to the target receptor DSG2. To demonstrate the versatility of JOC-*x*, we report here DSG2 binding and viral inhibition following conjugation to pegylated biotin, streptavidin-europium, DOTA-europium, and poly(I:C). We also demonstrated that JOC-poly(I:C) induces potent TLR3 activation by inducing >10-fold upregulation at concentrations that are ~625 fold lower than poly(I:C) alone. This effect is likely due to dual targeting and internalization of the conjugate, that is by JOC targeting the conjugate to the cells via DSG2 and activation of double stranded RNA sensors TLR3 and MDA5. We hypothesize that *in vivo* our JOC-poly(I:C) would likely exhibit enhanced potency by directing poly(I:C) to the tumor while at the same time minimizing non-specific activation of TLR3 receptors on healthy cells. Our JOC-*x* conjugates are primed for cGMP manufacture and hold much promise for upcoming validation and clinical studies.

## Methods

### Cloning and production of JO-1 derivatives

All genes used for this study were designed *in silico* using the software Serial Cloner v2.4.1. First generation JO derivatives, designated GB1–10, were based on the original JO-1 sequence as described previously^7,35^. The plasmid pET29a (Novagen) was linearized for Gibson cloning using outward facing primers pET29a_GB_UB (5′-TCCTCTCATATGTATATCTCCTTC) and pET29_GB_DT (5′-CTTAATTAGCTGAAATCACTAGT) using a 1/10000 dilution of plasmid as template in the PCR reaction. GB sequences were appended with nucleotides corresponding to the overlap regions of pET29a entry vector and were synthesized as gBlocks™ by Integrated DNA Technologies (Coralville, IA). Gibson cloning of gBlocks into the pET29a entry vector was performed using the Gibson Cloning Master Mix (New England Biolabs, Ipswich, MA) per manufacturer’s instructions. Cloned GBs were sequence confirmed (Genewiz, South Plainfield, NJ) and produced in *E. coli* Rosetta BL21 (DE3) (EMD Millipore, Darmstadt, Germany).

### rhDSG2 binding Western blot

One microgram of junction opener proteins or derivatives were resolved by non-reduced and non-boiled SDS-PAGE using 4–20% Tris-glycine gradient gels. Proteins were transferred to a polyvinylidene fluoride (PVDF) membrane and blocked with blocking buffer [1× TBS supplemented with 0.05% Tween-20 (TBST) and 10% nonfat dry milk (NFDM)] for 2 h at RT. Membranes were incubated with a 0.75 µg/mL solution of rhDSG2 (Leinco Technologies, Inc., Fenton, MO) in blocking buffer for 1 h at RT. Following three 10 min washes with TBST, the membrane was incubated with a 1/2000 dilution of murine anti-human DSG2 monoclonal Antibody 6D8 (Bio-Rad, Hercules, CA) in blocking buffer for 1 hr at RT. For detection, the membrane was incubated with a 1/2000 dilution of goat-anti-mouse conjugated to horseradish peroxidase (Southern Biotech, Birmingham, AL) in blocking buffer for 1 h at RT. After three washes with TBST and three washes with diH2O, bound HRP was detected using TMB Stabilized Substrate for Horseradish Peroxidase (Promega, Madison, WI).

### Viral inhibition assay

HeLa cell suspensions were confirmed to be >98% viable, their concentration adjusted to 1 × 10^5^ cells/ml, and then plated in 96 well plates with 200 µL per well (Corning, Inc., Corning, NY). Following 18 h incubation at 37 °C, 5% CO_2_, growth media was discarded and replaced with 62.5 µL of protein in complete Dulbecco’s Modified Eagles Medium (cDMEM) [cDMEM = DMEM supplemented with 10% Fetal Bovine Serum, 2 mM Glutamax, and 100 U/mL penicillin and 100 ug/mL Streptomycin (all components from Gibco)]. A total of 11 half-log dilutions were tested in quadruplicate for each protein. Following 1 h incubation, 50 µL of Ad3-GFP virus in complete DMEM was added at a Multiplicity of Infection (MOI) of 100. Two hours later, media was removed and replaced with fresh DMEM, and the plates incubated at 37 °C and 5% CO_2_. The following day, GFP fluorescence was measured from the bottom read orientation at 475 nm Excitation and 505 nm Emission using a SpectraMax i3x plate reader (Molecular Devices, Inc., Sunnyvale, CA). Data were plotted using Graphpad Prism 7 (GraphPad Software, Inc., La Jolla, CA) and IC_50_ values determined using a 5-parameter non-linear fit of the sigmoidal curves in Softmax Pro software (Molecular Devices).

### SPR analysis of binding affinities to rhDSG2

SPR analysis was performed at 25 °C using a Biacore 3000 instrument (GE Healthcare, Pittsburgh, PA). For immobilization of DSG2, rhDSG2 (10 μg/ml) was captured on a CM5 sensor chip using amine coupling chemistry until a coupling level of 6000 RU was obtained. GB7_V239D_ and GB3_V239D_ binding were measured in 10 mM HEPES, 150 mM NaCl, 0.005% surfactant P20, 2 mM CaCl_2_ pH 7.4 at a flow rate of 15 μl/min. A total of three dilutions of GB7_V239D_, ranging from 19 nM to 9.5 nM, and 4 dilutions of GB3_V239D_, ranging from 8.3 nM to 0.83 nM, were tested. For immobilization of junction opener proteins, JO-4 and GB7_V239D_ were diluted to 10 μg/ml in 10 mM sodium acetate pH 4.5 and immobilized on a CM5 sensor chip (approximately 6000 RU) by standard amine coupling chemistry. Human rhDSG2 binding was measured in 10 mM HEPES, 150 mM NaCl, 0.005% surfactant P20, 2 mM CaCl_2_ pH 7.4 at a flow rate of 15 μl/min. A total of 5 dilutions of rhDSG2, ranging from 17.4 nM to 0.87 nM, were tested in triplicate. The surfaces were regenerated by pulse injection of 5 mM EDTA. The signal recorded on a reference flow cell without protein was subtracted from those signals obtained on protein. For association data, proteins were injected at a flow rate of 15 μL/min for 180 seconds. Following the association phase, buffer was flowed at 15 μL/min for an additional 150 seconds to measure dissociation. Binding curves were analyzed using BIAEvaluation software (GE Healthcare) and data was fit to a 1:1 Langmuir interaction model. Affinities were mathematically derived by dividing the dissociation rate by the association rate.

### Size-exclusion chromatography and multi-angle light scattering analysis (SEC-MALS)

Size-exclusion chromatography was performed on an Agilent 1200 HPLC system (Agilent Technologies, Santa Clara, CA) with GE HealthCare Superdex 200 Increase 10/300 GL prepacked column and 1X TBS at 1 mL/min. JO-4, GB3_V239D_, and GB7_V239D_ were at concentrations of 2.5 mg/mL, 2.8 mg/mL, and 2.5 mg/mL in PBS with 5% glycerol, respectively, with 100 μL of each injected. Protein was measured via UV absorbance at 280 nm and sizes determined based on multi-angle light scattering (MALS), with concentration determined using differential refractive index. Analyses were performed using a miniDAWN TREOS MALS detector and Optilab T-rEX differential refractometer using ASTRA software (Wyatt Technology Corporation, Santa Barbara, CA), with the weighted average of the molecular weight reported in g/mol (Mw). For reduction of proteins, DTT was added to an initial concentration of 1.6 mM, proteins were incubated at 50 °C for 10 min, and then an additional 1.6 mM DTT was added to maintain the reduced conformations throughout the SEC-MALS analysis.

### Transmission electron microscopy

Recombinant GB proteins were visualized by negative-stain electron microscopy. Standard mica-carbon preparations were used with protein at 0.1 mg/ml. Samples were stained using 2% (wt/vol) uranyl acetate and visualized on a JEOL (JEM-1200EXII) electron microscope at 100 kV. Images were acquired and analyzed by digital micrograph software (Gatan).

### Conjugation of JOC-*x* to PEG-biotin or DOTA and labeling with europium

JOC-*x* was conjugated to both PEG_2_-biotin and PEG_11_-biotin using the EZLink^TM^ Maleimide-PEG2-Biotin and EZLink^TM^ Maleimide-PEG11-Biotin kits respectively (ThermoFisher Scientific, Waltham, MA, USA). JOC-x protein was first reduced by adding dithiothreitol (DTT) at a concentration of 10 mM and incubated at 37C for 1 hour. DTT was then removed by running the solution through Zeba desalting columns (ThermoFisher Scientific, Waltham, MA, USA). The maleimide-PEG_2/11_-Biotin was then added at a molar ratio of 20:1 (biotin:JOC-*x*) and allowed to react, rotating for 24 hours at room temperature. Excess PEG_2/11_-Biotin was then removed, again by Zeba desalting columns. Confirmation of biotin conjugation was subsequently carried out by SDS-PAGE analysis. LanthaScreen^TM^ Eu-Streptavidin (ThermoFisher Scientific, Waltham, MA, USA) was used as the streptavidin-europium (SA-Eu) payload. JOC-Biotin and the SA-Eu were mixed at a molar ratio of 1:1 in solution, allowed to bind at room temperature for 30 minutes, and then were subsequently run on a SDS-PAGE gel. The gel was then transferred to a PVDF membrane and imaged using the SpectraMax i3x imager (Molecular Devices, San Jose, CA, USA), at an emission wavelength of 616 nm.

For DOTA conjugation to JOC-*x*, JOC-*x* was reduced by adding DTT at a concentration of 0.1 µg/mL and incubated at 37 °C for 1 hour. DTT was then removed by running the solution through Zeba desalting columns. Maleimido-mono-amide-DOTA (Macrocyclics, Dallas, TX, USA) was then added to JOC-x at a molar ratio of 10:1 (DOTA:JOC-x), and allowed to react at room temperature overnight. Confirmation of DOTA conjugation to JOC-x was carried out by SDS-PAGE and mass spectrometry. Europium ions were sourced from EuCl_2_·6H_2_O (Sigma-Aldrich, St. Louis, MO, USA). The JOC-DOTA was buffer exchanged into an ammonium acetate buffer at pH 5.8 to promote chelation of Eu. EuCl_2_·6H_2_O was then added at a molar ratio of 10:1 (EuCl_2_·6H_2_O:JOC-DOTA) and allowed to react at 37 °C for 1 hour and left at room temperature for 24 hours. 10 × molar excess (of Eu added) of ethylenediaminetetraacetic acid (EDTA) was then added to chelate and remove free Eu. This was followed by running the solution through a Zeba desalting column (ThermoFisher Scientific, Waltham, MA, USA) to eliminate residual europium and to restore the protein buffer to a phosphate pH 7.0 buffer. Confirmation of Eu chelation was done by resolving the proteins on SDS-PAGE, transferring to PVDF membrane, and imaging phosphorescence using a SpectraMax i3X (Molecular Devices, San Jose, CA, USA) at an emission wavelength of 616 nm.

### Conjugation of JOC-x to poly(I:C) and measurement of TLR3 signaling

Low molecular weight (LMW) poly(I:C) (Invivogen) was resuspended to a final concentration of 10 mg/mL in 10 mM sodium phosphate, 150 mM NaCl, 10 mM EDTA, pH 7.2. Next, 0.75 mL of LMW poly(I:C) was mixed with 125 mg of 1-ethyl-3-(3-dimethylaminopropyl)carbodiimide (EDC) in a 15-mL Falcon tube. Finally, 0.5 mL of 250 mM ethylenediamine in 100 mM imidazole was added to the poly(I:C), thoroughly vortexed, and 2 mL of 100 mM imidazole was added and allowed to react overnight at RT. The following day, unreacted EDC and ethylenediamine were removed from the poly(I:C)-NH2 by two passes through 5 mL 7k MWCO Zeba^TM^ Spin Desalting Columns (Thermo Scientific) that were equilibrated with 100 mM sodium phosphate, 150 mM NaCl, 5 mM EDTA, pH 7.2. Purified poly(I:C)-NH2 was then incubated with a 100-fold molar excess of sulfosuccinimidyl 4-(N-maleimidophenyl)butyrate (sulfo-SMPB) (Thermo Scientific) for 1 hour at RT before removing excess sulfo-SMPB with two passes through Zeba™ columns as described above. Simultaneously, JOC-*x* was incubated with 5 mM tris(2-carboxyethyl)phosphine (TCEP) for 30 minutes at RT before removing excess TCEP with two passes through Zeba^TM^ columns. Finally, poly(I:C)-NH2 was incubated with JOC-*x* at a 1:2 molar ratio at RT overnight. Conjugation was confirmed by nucleic acid gel electrophoresis, SDS-PAGE, and Western blot.

TLR3 signaling was measured using a commercial HEK-Blue™ hTLR3 Assay (Invivogen, San Diego, CA) according to manufacturer’s specifications. TLR3 activation resulted in NFκB- and AP1-induced production of the reporter gene Secreted Embryonic Alkaline Phosphatase (SEAP).

## Supplementary information

Supplementary Information

## Data Availability

Upon publication of the manuscript, we will provide reasonable amounts of requested material following execution of a Material Transfer Agreement for the purposes of replicating data in the paper. The junction opener technology in this manuscript is protected by patents held by University of Washington.
