# Supplementary material for: Structure-based Design of JOC-x, a Conjugatable Tumor Tight Junction Opener to Enhance Cancer Therapy
Source: Sci Rep. 2019 Apr 16;9:6169. doi: 10.1038/s41598-019-42229-3 (PMC6467980; doi:10.1038/s41598-019-42229-3)
Supplement: Supplementary file 1 — Supplementary Information [file 41598_2019_42229_MOESM1_ESM.pdf]

Supplementary Figures

STRUCTURE-BASED DESIGN OF JOC-X, A CONJUGATABLE TUMOR TIGHT JUNCTION OPENER TO ENHANCE CANCER THERAPY

Ragan Pitner[1,2+], Jiho Kim[1,3+], Jenn Davis-Bergthold[1], Cheri Turner[1], Emilie Vassal-Stermann[4], Hongjie Wang[5], Jaclyn Adams[5], Lauren Carter[6], Jeffrey A. Ahlgren[7], Pascal Fender[4], André Lieber[5,8], Darrick Carter[1,3,5,8], and Sean A. Gray[1*]

[1]PAI Life Sciences, Inc., Seattle, WA
[2]University of Washington, Dept. of Immunology, Seattle, WA
[3]University of Washington, Program in Pathobiology, Dept. of Global Health, Seattle, WA
[4]Institut de Biologie Structurale, UMR5075, CNRS/CEA/UGA, Grenoble, France
[5]University of Washington, Division of Medical Genetics, Seattle, WA
[6]Institute for Protein Design, Department of Biochemistry, University of Washington, Seattle, WA
[7]Wyatt Technology Corporation, Santa Barbara, CA
[8]Compliment Corp., Seattle, WA

[+]These authors contributed equally to this work
[*]Correspondence and requests for materials should be addressed to SAG (sean.gray@pailifesciences.com)

24 **Supplementary Figure 1**

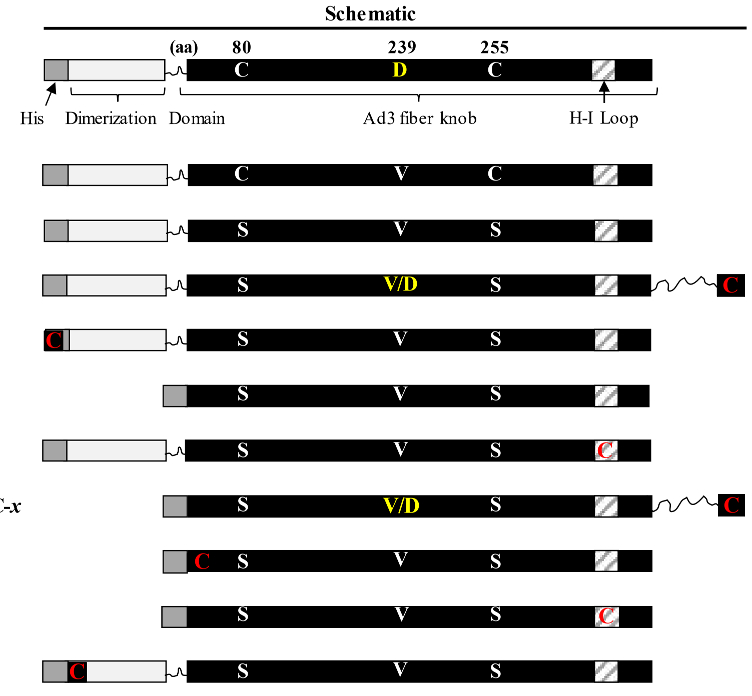

Designation	V239D mutation?	Dimerization domain?	Wild-type cysteines	Location of newly inserted cysteine?
JO-4	Yes	Yes	Yes	No
GB1	No	Yes	Yes	No
GB2	No	Yes	No	No
GB3	With and without	Yes	No	Following C-terminal G4S linker
GB4	No	Yes	No	Preceding 6*x*-his tag
GB5 (ΔDD)	No	No	No	No
GB6	No	Yes	No	Within the H-I loop
**GB7 (ΔDD)/JOC-*x***	With and without	No	No	Following C-terminal G4S linker
GB8 (ΔDD)	No	No	No	Following 6*x*-his tag
GB9 (ΔDD)	No	No	No	Within the H-I loop
GB10	No	Yes	No	Following 6*x*-his tag within dimerization domain

Supplementary Figure 1. Schematic representation of JO-derived protein constructs. On the top is a crude representation of the affinity enhanced JO-4 protein with key features indicated below. Letters and numbers indicate locations and specific amino acids that were altered for this analysis. The affinity enhancing V239D mutation (V or D) is shown in yellow type, native cysteines (C) are shown in white type, serines introduced by mutation of native cysteines are shown as (S) in white type, and newly inserted cysteines for directed conjugation are shown as (C) in red type. Only GB3 and GB7 were constructed and analyzed with both the low affinity valine (V) and high-affinity aspartic acid (D) mutations. Key changes to each protein construct are summarized by the table to the right of the pictoral image.

Supplementary Figure 2

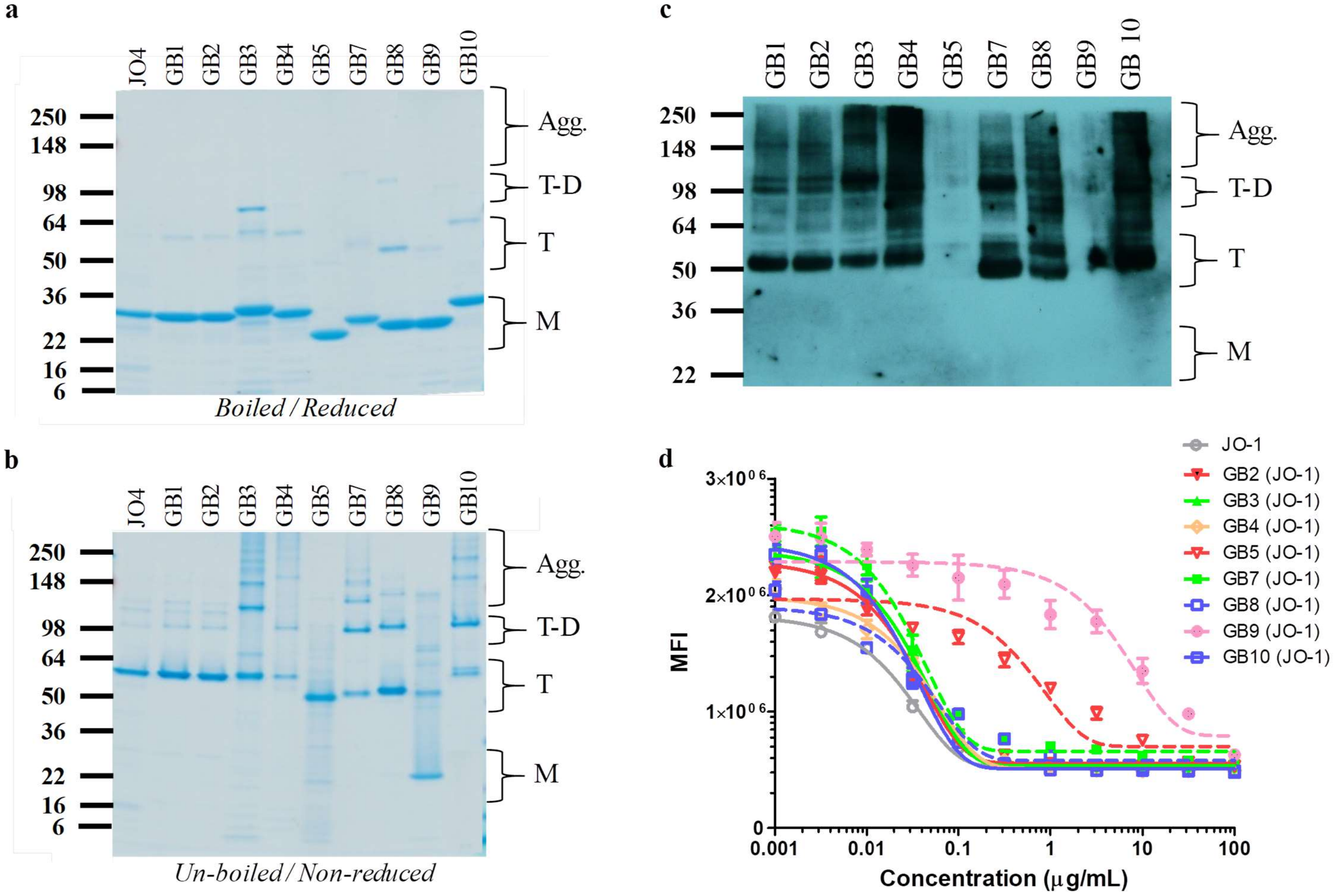

Supplementary Figure 2. Production and characterization of JO-1 derived proteins. Panels (a) shows the boiled and reduced analysis of the GB proteins on Coomassie stained SDS-PAGE. **Panel (b)** shows the non-reduced and un-boiled analysis of the same proteins. **Panel (c)** shows a DSG2 binding Western blot of the GB proteins. In panels a, b, and c the multimeric forms are indicated to the right of the gel/blot whereby M= monomers, T= trimers, T-D= trimer-dimers, and Agg.= aggregates/multimers. **Panel (d)** shows the viral inhibition curves for each protein. The IC_{50} values are shown in Table 1.

Supplementary Figure 3

a

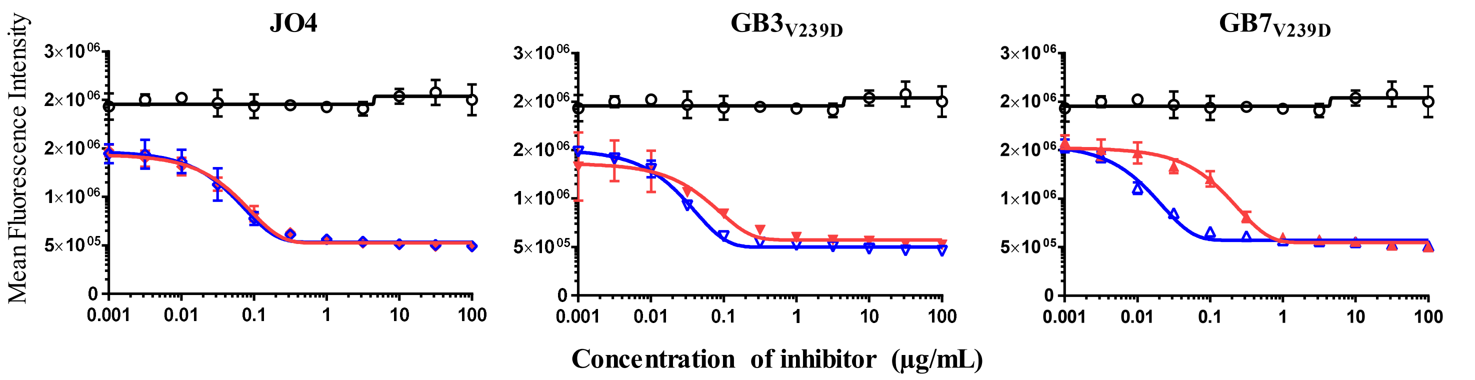

b

Protein	IC_{50} (µg/mL)		Fold lower activity upon reduction
	Oxidized	Reduced	
JO4	0.065	0.054	(0.8)
$GB3_{V239D}$	0.028	0.055	2.0
$GB7_{V239D}$	0.029	0.150	5.0

Supplementary Figure 3. Viral inhibition mediated by reduced and non-reduced proteins. Panel (a) shows the viral inhibition curves for reduced (red lines) and non-reduced (blue lines) JO-4, $GB3_{V239D}$, and $GB7_{V239D}$ proteins. Black lines show the VIA curves for the BSA control. **Panel (b)** reports the IC_{50} values for each of the VIA curves approximating the fold change in viral inhibition activity following reduction.
